# Supplementary material for: Computational Pipeline for Reference-Free Comparative Analysis of RNA 3D Structures Applied to SARS-CoV-2 UTR Models
Source: Int J Mol Sci. 2022 Aug 25;23(17):9630. doi: 10.3390/ijms23179630 (PMC9455975; doi:10.3390/ijms23179630)
Supplement: Supplementary file 1 [file ijms-23-09630-s001.zip › Supplementary_Notes_And_Figures_SARS_COV_2.pdf]

Supplementary Notes

Input RNA sequences for the 3D modeling

The extended 293-nucleotide sequence of 5’ untranslated region (5'-UTR):

5'-
AUUAAAGGUUUAUACCUUCCCAGGUAACAAACCAACCAACUUUCGAUCUCUUG
UAGAUCUGUUCUCUAAACGAACUUUAAAAUCUGUGUGGCUGUCACUCGGCUG
CAUGCUUAGUGCACUCACGCAGUAUAAUUAAUAACUAAUUACUGUCGUUGACA
GGACACGAGUAACUCGUCUAUCUUCUGCAGGCUGCUUACGGUUUCGUCCGU
GUUGCAGCCGAUCAUCAGCACAUCUAGGUUUCGUCCGGGUGUGACCGAAAG
GUAAGAUGGAGAGCCUUGUCCCUGGUUUCAACG -3'

The sequence of 3'-UTR was the following (337 nt):

5'-
ACUCAUGCAGACCACACAAGGCAGAUGGGCUAUAUAAACGUUUUCGCUUUUC
CGUUUACGAUAUAUAGUCUACUCUUGUGCAGAAUGAAUUCUCGUAACUACAU
AGCACAAGUAGAUGUAGUUAACUUUAAUCUCACAUAGCAAUCUUUAAUCAGUG
UGUAACAUUAGGGAGGACUUGAAAGAGCCACCACAUUUUCACCGAGGCCACG
CGGAGUACGAUCGAGUGUACAGUGAACAAUGCUAGGGAGAGCUGCCUAUAUG
GAAGAGCCCUAAUGUGUAAAAUUAAUUUUAGUAGUGCUAUCCCCAUGUGAUU
UUAAUAGCUUCUUAGGAGAAUGAC-3'

The prediction methods

Bujnicki group

The Bujnicki group adopted a simple approach, which involved folding of the SARS-CoV-2 5' and 3' terminal regions using the SimRNA method (Boniecki et al. 2016). In the case of the 5’-UTR, this region was modeled together with the beginning of the ORF1, thus, part of this coding sequence was taken into account for modeling (residues 1-293). On the other hand, the 3’-UTR was modeled as the 337 residues mentioned above (positions 29531-29867). Secondary structure predictions reported for various coronaviruses as well as those obtained for SARS-CoV-2 and its homologs, were taken into account to derive the secondary structure restrains used for modeling (Manfredonia et al. 2020; Rangan et al. 2021; Rangan et al. 2020). A secondary structure-based sequence alignment was generated for the UTR sequences based on the combination of various automated methods and visual analyses. 2D restraints were

extracted for regions where a structural consensus was found according to the above-mentioned predictions; while SimRNA was enabled to identify the preferred base-pairing patterns for the regions where no restraints were specified due to the lack of consensus. Following, the simulation run with default parameters, 1% of the best-scored 3D models were extracted and clustered at the threshold of 15 Å. Representative members of the five largest clusters were selected for submission as preliminary models. At this initial stage, no refinement of the models was attempted.

Chen Group

The Chen group used a hierarchical approach to predict 3D structures of both the 5'- and the 3'-UTRs. At the first stage, sequence-based secondary structure prediction was conducted. Next, the corresponding 3D structures were calculated with the secondary structures data as constraints. In the case of secondary structure prediction, the free energy-based Vfold2D model was used (Cao and Chen 2005; Cao and Chen 2006; Cao and Chen 2009; Xu et al. 2014; Xu and Chen 2015; Zhao et al. 2017). Vfold2D algorithm computed motif-based loop entropies and enumerated all the possible intra-loop mismatches for the free energy calculations. The entropies of pseudoknot loops were determined from the probability of loop closure. As a result, the optimal secondary structure and an ensemble of suboptimal RNA secondary structures ranked by free energies were obtained. Additionally, in order to select secondary structures for the SARS-CoV-2 3'-UTR, information such as pseudoknotted loops identified in coronaviruses (Williams et al. 1999; Goebel et al. 2004) was taken into account. The 3D structure prediction was performed using IsRNA-Vfold3D (Zhao et al. 2017; Zhang et al. 2018), a coarse-grained molecular dynamics (CGMD) simulation method. In the IsRNA CGMD model, four or five beads were used to represent the pyrimidine and purine ribonucleotides, respectively, and an accurate coarse-grained force field was extracted from the RNA iterative reference state simulations. In the case of a given secondary structure and sequence, the system assembled motif-based templates to build the initial structure for CGMD. For each secondary structure constraint, 100-ns CGMD simulations were performed followed by the conformational clustering. Finally, the predicted 3D coarse-grained structures were transformed into all-atom conformations, which were further refined using AMBER energy minimization.

Ding group

The Ding group used a similar multi-scale discrete molecular dynamics (DMD)-based RNA modelling approach as described in (Miao et al. 2020). In short, Coarse-Grained (CG) RNA simulations using replica exchange DMD in case of each sequence were applied and the representative low-energy states obtained by cluster analysis for all-atom reconstruction were selected. Additionally, consensus secondary structures obtained from secondary structure predictions (from RNAstructure (Xu and Mathews 2016), Rfam or SHAPE data) and bioinformatics analysis were included as constraints in CG simulations (Miao et al. 2020).

Szachniuk group

Szachniuk group applied RNAComposer ((Popenda et al. 2012; Purzycka et al. 2015; Antczak et al. 2016) to predict the 3D structures for the input sequences, namely 268nt for 5'-UTR and 337nt for 3'-UTR. Computation started from running eight RNAComposer-integrated algorithms for RNA secondary structure prediction: RNAstructure (Reuter and Mathews 2010), RNAfold (Langdon et al. 2018), Contrafold (Do et al. 2006), ContextFold (Zakov et al. 2011), Centroid_fold (Sato et al. 2009), Ipknot (Sato et al. 2011), RNAshapes (Steffen et al. 2006), and HotKnots (Ren et al. 2005). Additionally, RNAalifold (Lorenz et al. 2011) and CentroidAlifold (Hamada et al. 2011) were used to derive consensus secondary structures from precomputed multiple alignments of homologous RNA sequences. Sequence alignment was obtained from BLAST (Altschul et al. 1990) and NeoBio (de Carvalho 2003) applied on a set of 18 sequences provided as additional data for this puzzle. In the latter case, we restricted the set to five 5'-UTR and nine 3'-UTR sequences, which showed at least 90% similarity to the target sequence. For 5'-UTR, 20 secondary structures with the lowest energy obtained with RNAstructure (energy between -81.2 and 77.1 kcal/mol) were considered - here, the sequence for processing was extended to 296 and 300nts. We analysed the predicted 2D structures with respect to the number of domains. Based on this study and the literature review (Yang and Leibowitz 2015), we selected a set of secondary structures for further processing. For each of them, RNAComposer predicted up to 100 3D structure models. These 3D models were automatically evaluated according to all RNA-Puzzles measures computed by RNA QUality Assessment tool (RNAQUA) (Magnus et al. 2020). Five best RNA 3D models were selected for each considered secondary structure. All of them were checked for entanglements (Popenda et a. 2021) and stereochemical errors and those that passed the test, were ranked according to the total energy. The final choice of models for submission was based on expert decision.

Supplementary Figures

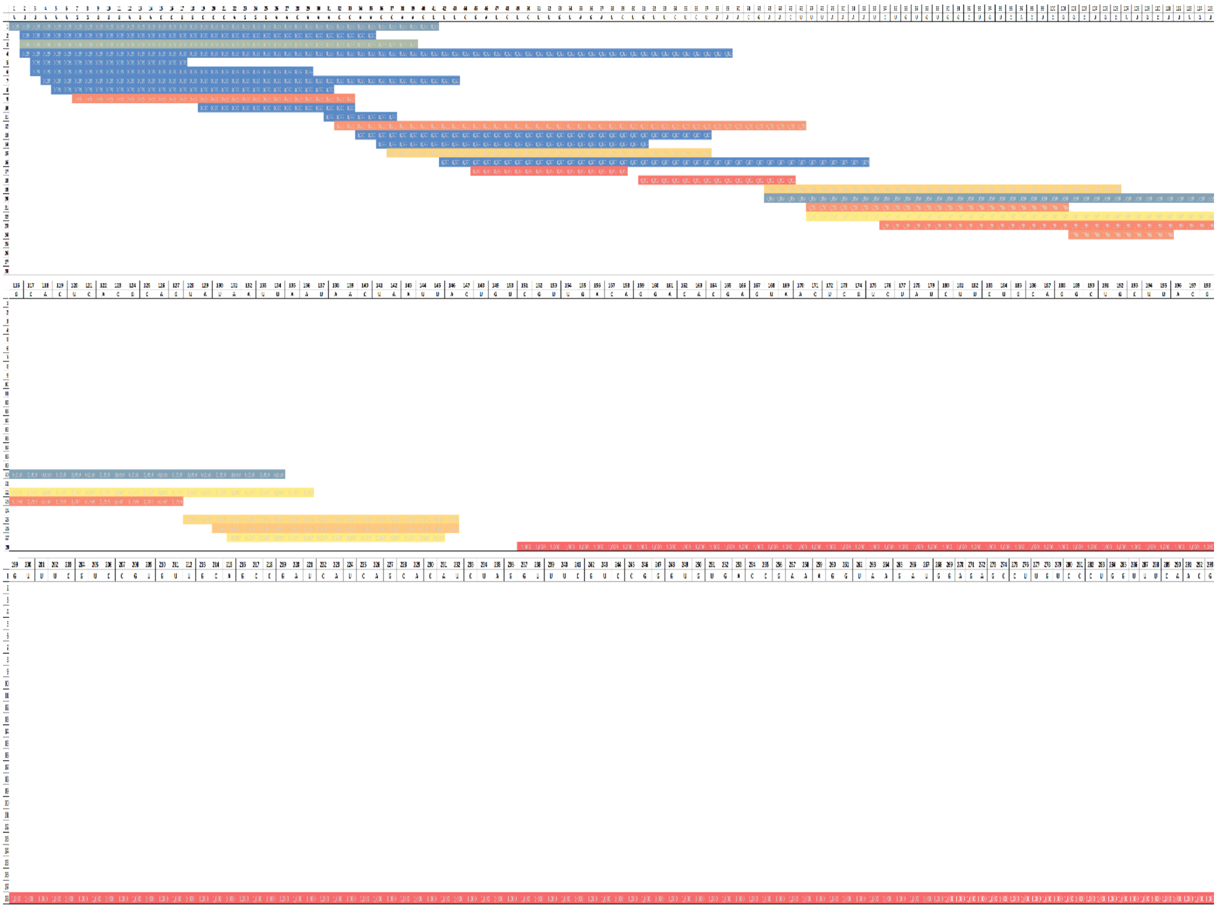

Figure S1. Diagram representing the distribution of the potential and conserved RNA 2D domains within 5'-UTR region, grouped by RNA sequence. The results were obtained for all secondary structures derived from 3D models cut to the size of 293 nt. Red coloured bars correspond to the elements present in over 40% of models, yellow ones to the fragments preserved in at least two models and blue to those that were found in only one model.

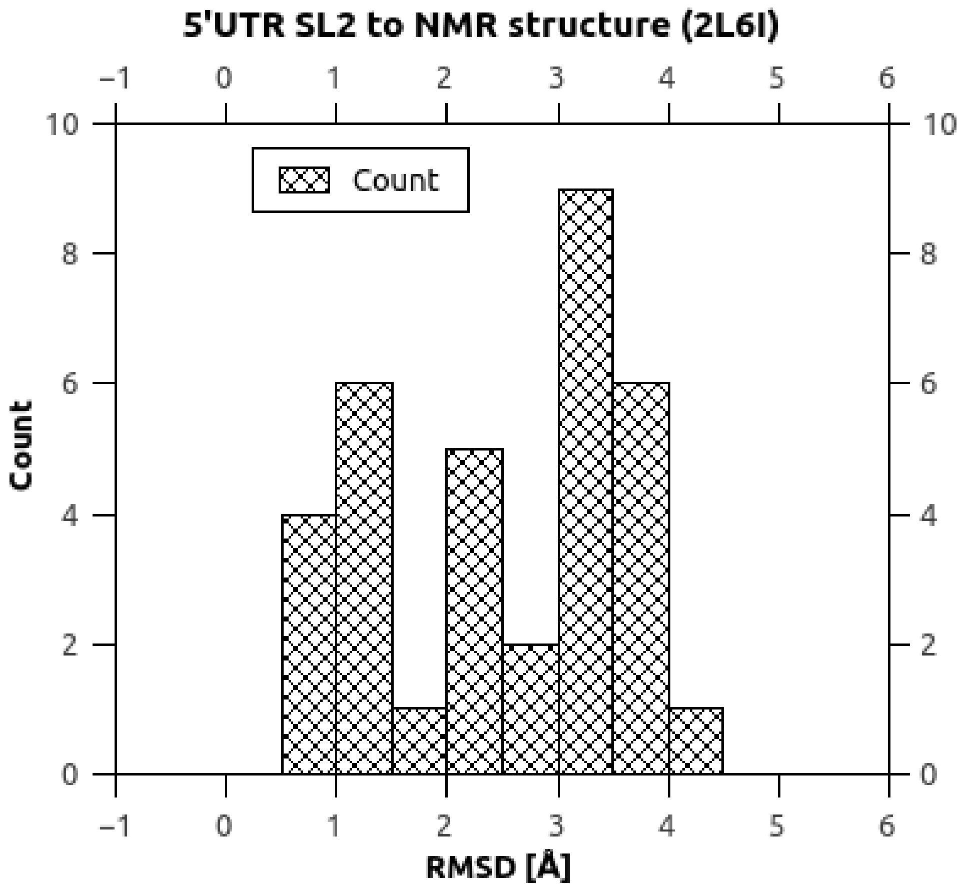

Figure S2. Diagram representing the distribution of RNA 3D models of SARS-CoV-2 5'-UTR that included SL2 domain, grouped by their RMSD distance to the three-dimensional crystal structure of SL2 solved for the SARS-CoV-1 virus genome.

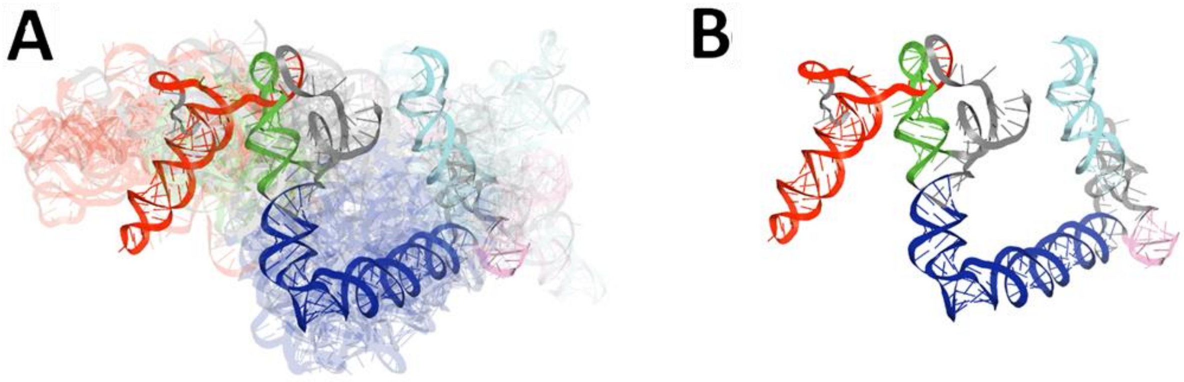

Figure S3. Visualization of the results of global RMSD-based analysis and comparison of RNA 3D models for 3'-UTR region of SARS-CoV-2. Domains are coloured as follows: BSL (red), P2 (green), Octa (light purple), SLM (cyan), HVR stem (blue). The centroid of the ensemble is depicted in each case in solid colours while the remaining ensemble members are shown as transparent structures. (A) The ensemble of 3D RNA pseudoknotted structures and (B) the centroid of this ensemble.

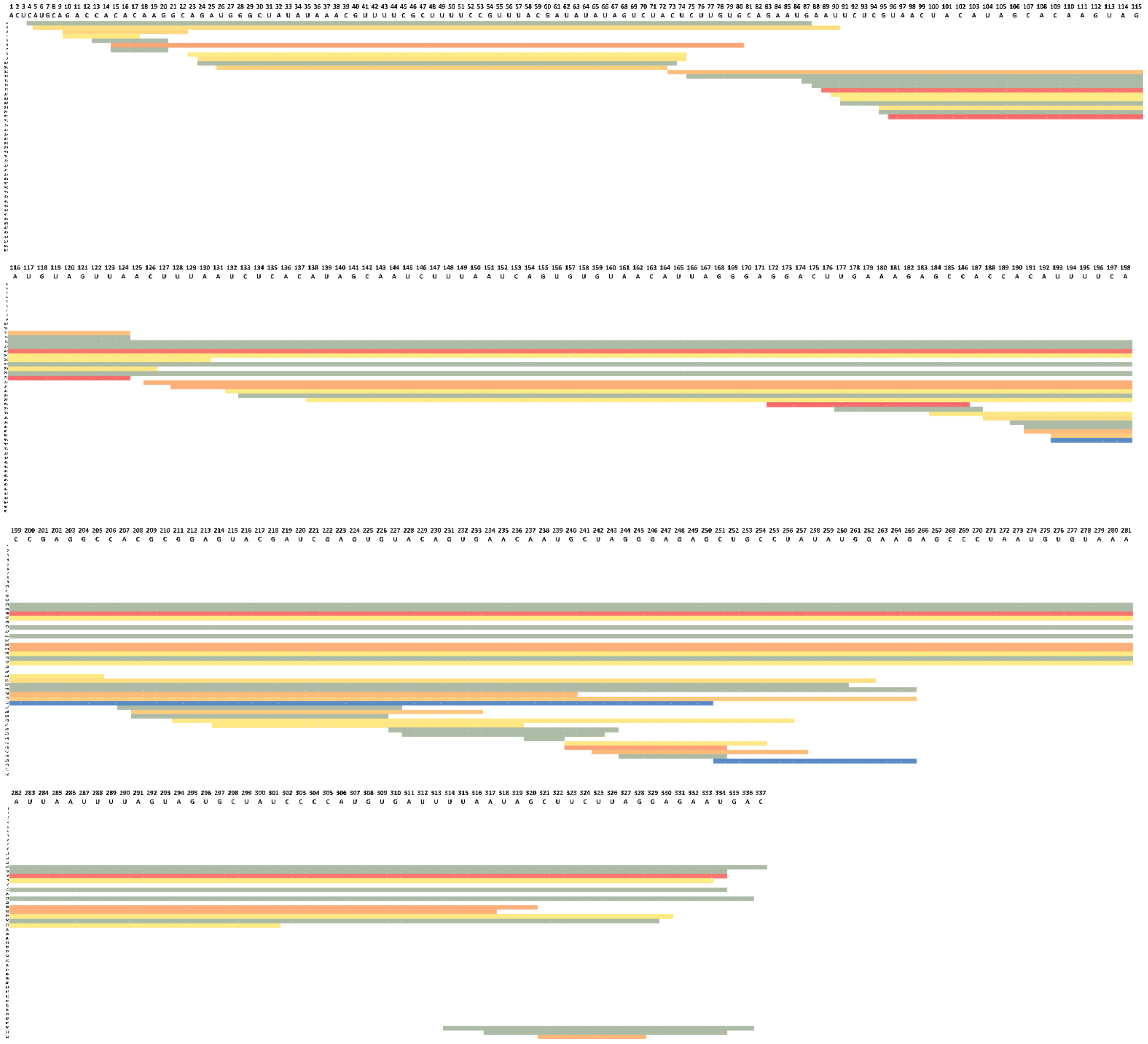

Figure S4. Diagram representing the distribution of the potential and conserved RNA 2D domains within the 3'-UTR region, grouped by RNA sequence. Red-colored bars correspond to the elements present in over 40\% of models, yellow ones to the fragments preserved in at least two models and blue to those found in one model.

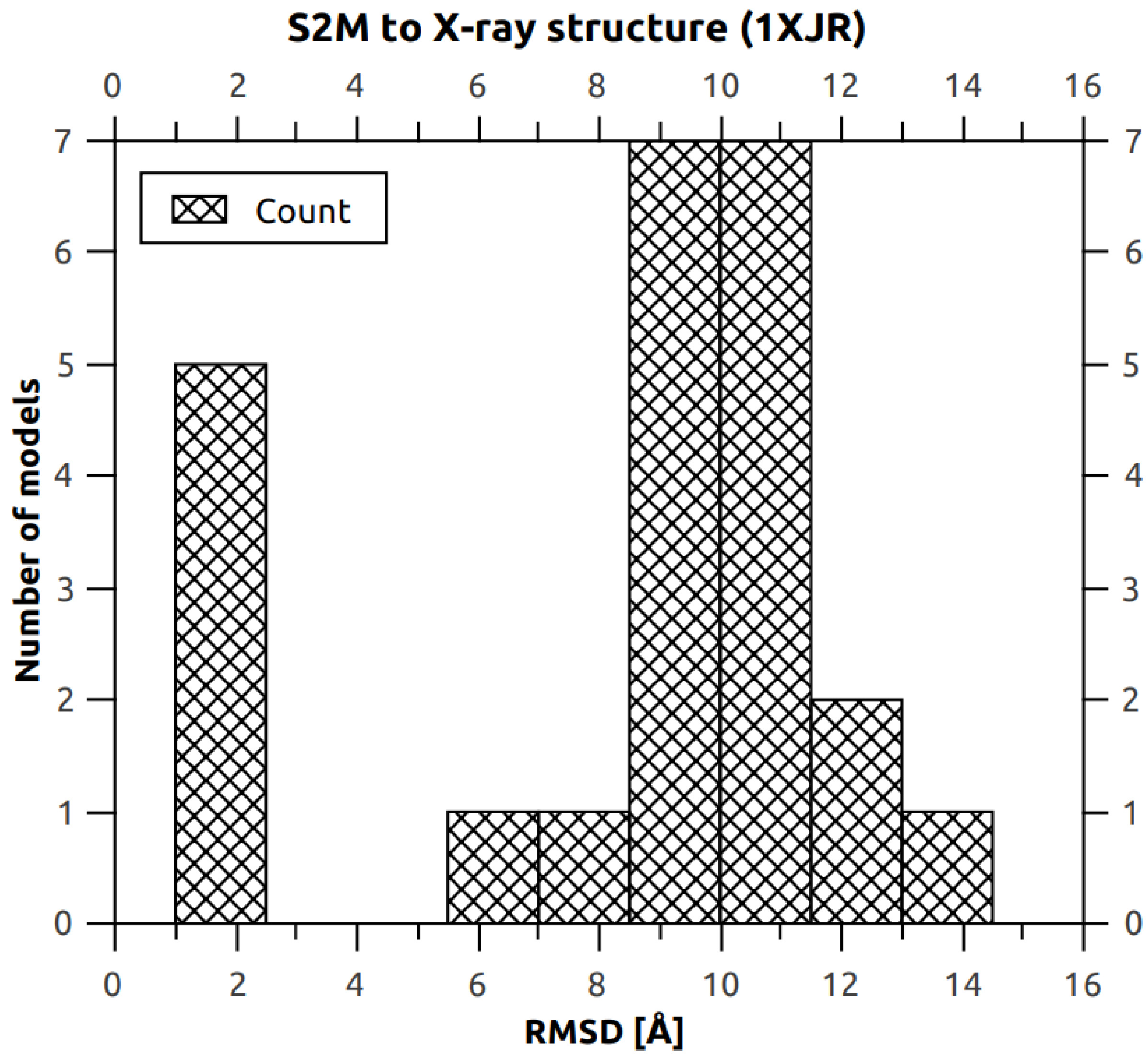

Figure S5. Diagram representing the distribution of RNA 3D models of SARS-CoV-2 3'-UTR that included s2m domain, grouped by their RMSD distance to the three-dimensional crystal structure of s2m solved for the SARS-CoV-1 virus genome.
