# Supplementary material for: Computational Pipeline for Reference-Free Comparative Analysis of RNA 3D Structures Applied to SARS-CoV-2 UTR Models
Source: Int J Mol Sci. 2022 Aug 25;23(17):9630. doi: 10.3390/ijms23179630 (PMC9455975; doi:10.3390/ijms23179630)
Supplement: Supplementary file 1 [file ijms-23-09630-s001.zip › Table_S2a.pdf]

Table S2a. RNA 3D models of SARS-CoV-2 5'-UTR with entanglements. For each model, the types of entanglements are given.

3D RNA model	Simple entanglements	Higher-order entanglements
5UTR		
5UTR-Bujnicki-02.pdb		L(D), L(S)
5UTR-Bujnicki-05.pdb		L(D)
