# Supplementary material for: Computational Pipeline for Reference-Free Comparative Analysis of RNA 3D Structures Applied to SARS-CoV-2 UTR Models
Source: Int J Mol Sci. 2022 Aug 25;23(17):9630. doi: 10.3390/ijms23179630 (PMC9455975; doi:10.3390/ijms23179630)
Supplement: Supplementary file 1 [file ijms-23-09630-s001.zip › Table_S2b.pdf]

Table S2b. RNA 3D models of SARS-CoV-2 3'-UTR with entanglements. For each model, the types of entanglements are given.

**3D RNA model**	**Simple entanglements**	**Higher-order entanglements**
3UTR		
no_PK		
3UTR-Bujnicki-03.pdb	L(L), L(D)	
3UTR-Bujnicki-04.pdb	L(L), L(D)	
3UTR-Bujnicki-05.pdb	L(L), L(D) , L(D)	
3UTR-Ding_7.pdb	L(L) , L(D)	
PK		
3UTR-Chen-2_1.pdb		L(S)
3UTR-Chen-2_2.pdb	L(S)	L(S)
3UTR-Chen-2_3.pdb	L(S)	L(S)
3UTR-Chen-2_4.pdb	L(S)	L(S)
3UTR-Chen-2_5.pdb	L(S)	L(S)
3UTR-Das-01_1.pdb	L(S)	L(S)
3UTR-Das-02_1.pdb	L(S)	L(S)
3UTR-Das-03_1.pdb		L(D), L(D), L(S)
3UTR-Das-04_1.pdb	L(S)	L(S)
3UTR-Das-05_1.pdb	L(S)	L(S)
3UTR-Das-06_1.pdb	L(S)	L(S)
3UTR-Das-07_1.pdb	L(S)	L(S)
3UTR-Das-08_1.pdb	L(S)	L(S)
3UTR-Das-09_1.pdb	L(S)	L(S)
3UTR-Das-10_1.pdb	L(S)	L(S)
